# Chunking of Control: An Unrecognized Aspect of Cognitive Resource Limits

**DOI:** 10.5334/joc.275

**Published:** 2023-05-04

**Authors:** Ausaf A. Farooqui, Tamer Gezici, Tom Manly

**Affiliations:** 1 Bilkent University, Turkey; 2 National Magnetic Resonance Research Center, Turkey; 3 MRC-Cognition & Brain Sciences Unit, UK; 4 University of Cambridge, UK

**Keywords:** Cognitive Limits, Programs, Chunking, Hierarchical Execution

## Abstract

Why do we divide (‘chunk’) long tasks into a series of shorter subtasks? A popular view is that limits in working memory (WM) prevent us from simultaneously maintaining all task relevant information in mind. We therefore chunk the task into smaller units so that we only maintain information in WM that is relevant to the current unit. In contrast to this view, we show that long tasks that are not constrained by WM limits are nonetheless chunked into smaller units. Participants executed long sequences of standalone but demanding trials that were not linked to any WM representation and whose execution was not constrained by how much information could be simultaneously held in WM. Using signs well-known to reflect beginning of new task units, we show that such trial sequences were not executed as a single task unit but were spontaneously chunked and executed as series smaller units. We also found that sequences made of easier trials were executed as longer task units and vice-versa, further suggesting that the length of task executed as one unit may be constrained by cognitive limits *other* than WM. Cognitive limits are typically seen to constrain how many things can be done *simultaneously* e.g., how many events can be maintained in WM or attended at the same time. We show a new aspect of these limits that constrains the length of behaviour that can be executed *sequentially* as a single task-unit.

## Introduction

We often describe tasks at the level of goals rather than at the series of executed steps. Hence, we say we are going to ‘make scrambled eggs’ rather than, ‘find bowl, find eggs, crack shells…”. An interesting question is whether and how, in addition to linguistic brevity, the intention to ‘make scrambled eggs’ itself tees up these component steps that are then executed as an unfolding program. Whilst ‘action lapses’ (we find ourselves reaching for cheese in the fridge out of habit) and sequencing errors (starting to whisk before the shell fragment is removed) occur, for the most part, these goal-directed sequences are executed in an efficient manner that considers many variations in the precise motor commands etc. needed to achieve each step. In what sense do the multiple flexible events making up a task correspond to one entity at the level of preparation/execution?

### Hierarchical Control Structures

Past studies have addressed this question using *predictable* tasks where the identity and sequence of component acts were accessible as a single representational structure. These structures have been variously termed plans, scripts, schemas or frames (Balaguer et al., 2016; Miller et al., 1960; Minsky, 1974; Norman & Shallice, 1986; Rumelhart & Norman, 1982; Schank & Abelson, 1977; Tomov et al., 2020). Thus, the script for ‘visiting a restaurant’ may contain sequentially organized subscripts for ‘entering a restaurant’, ‘waiting for a table’, ‘ordering food’, each of which may consist of more than one sequentially organized sub-subscripts (Bower et al., 1979). These structures are hierarchical in that they consist of representations at multiple levels with an asymmetric relation between them such that the lower-level, step-related representations can only be accessed by first accessing the higher-level, task-related representations (Broadbent, 1977; Schneider & Logan, 2007).

In related experiments, participants executed extended sequence of actions whose representations were hierarchically organized and accessible as a single mnemonic structure. These have included participants executing a practiced sequence of motor acts, speaking words or sentences of various lengths, and recalling memorized lists (Anderson et al., 1998; Dezfouli et al., 2014; Kahana & Jacobs, 2000; Lien & Ruthruff, 2004; Rosenbaum et al., 1983; Schneider & Logan, 2006). Despite variations across these experiments, they all show three common characteristics of hierarchical execution: (1) The first step that begins the sequence has a higher reaction time (RT) than subsequent steps, (2) this step 1 RT is longer at the beginning of longer/more complex sequences, (3) the behavioral cost of switching the item being executed (i.e. switch cost; Monsell, 2003) occurs only when this switch occurs within the sequence, and, crucially, not when it occurs across sequence boundaries.

Thus, when participants are given a sequence like CCSS to iteratively execute, where C and S stand for color and shape decisions to be made across sequential trials, then item 1 RT is the highest and correlates with the number of item-level switches in the sequence (Desrochers et al., 2015; Schneider & Logan, 2006). Hence item 1 RT is longer for a sequence like CSCS (with three item-level switches) than for a sequence like CCSS (with one item-level switch). These suggest that some control structure related to the whole sequence needs to be instantiated at the beginning of sequence execution. The control structure will be bigger/more complex for longer/more complex sequences, and hence the longer item 1 RT (Mayr, 2009; Perlman et al., 2010; Schneider & Logan, 2006, 2015). Once this is in place, subsequent steps require accessing item representations already in working memory (WM), hence their shorter RT. It is well-known that changing the rule being used to select the correct action (or rule-switch) leads to increased RT and error rates on the trial where this rule-switch occurs. This switch cost is specifically absent when rule-switch occurs across successive items belonging to different sequences (e.g., when a sequence like CCSS is iteratively executed – CCSS-CCSS… – step 1 involves a color to shape switch but shows no switch-cost; Schneider & Logan, 2006, 2015), suggesting that the change of the higher, sequence-level control structure at sequence boundaries also resets the lower-level trial-related rule configurations (Lien & Ruthruff, 2004).

These studies not only suggested that task-sequence representations control the identity and sequence of component acts/steps, but also suggested a key role of WM in this sequential organization of behavior; the higher-level control structures are maintained in WM as they control sequential behavior (O’Reilly & Frank, 2006). Here, WM is understood as the ability to maintain representations, objects, images, symbols during task executions (Baddeley, 2012; Cowan, 2016). Crucially, these studies have also suggested that WM limitations may limit the length of tasks that can be executed as one unit. During long memorized sequence executions, all items cannot be maintained in WM, so long sequences must be parsed into smaller chunks and executed piecemeal. Items related to the first chunk are maintained in WM and executed, then items related to the second chunk are put in WM and executed, and so on.

This was evident in Logan (2004). Here, participants first memorized a series of operations that could be applied to numbers. The first operation might be ‘high–low’ (press right if the number is greater than 5, left if lower), the next ‘Odd–Even’ (press right if the number is even, left if it is odd), the next ‘Digit–Word’ (press right if the number is a digit, left if it is a word) and so on. They were then asked to apply these operations, in order, to a series of digit/number words. When the participants began these sequences, the first trial showed an elevated RT consistent with loading the program from memory, followed by relatively low RTs on trials 2 and 3, that were presumably beneficiaries of that trial 1 preparation. However, subsequent peaks in RT were also observed at the 4th and 7th trials in the sequence. Logan (2004), inferred that whilst longer sequences of the correct task order were in memory, the participants could only develop a plan – a ‘chunk’ – for 3 trials at a time due to WM limits (see Anderson & Matessa, 1997; Bower, 1970; Kahana & Jacobs, 2000; Tulving, 1962 for similar findings). Switch cost tends to be reduced at these chunk points (Schneider & Logan, 2015), suggesting that beginning of these chunks involved removal of the old and instantiation of new higher-level control structure.

### Rethinking the Nature of Hierarchical Control Structures

So far, we have discussed instances in which participants knew in advance the precise sequence of actions, rules etc. that they would need to plan to execute over the coming task – indeed the ‘goal’ in many of these studies consisted only of the correctly ordered completion of the steps. Hierarchical control in such situations seems to be about specifying the identity and sequence of component steps. But many of our everyday tasks do not have a predictable sequence of steps. Nonetheless, their corresponding behaviors are reliably identified by the doer as one task despite consisting of these multiple variable steps (Vallacher & Wegner, 1987). Hierarchical control structures in these tasks, if present, cannot operate by specifying the identity and sequence of steps. Furthermore, even apparently routine tasks often require much flexibility in the particular sequence of actions required. We might, for example, first find the saucepan and then locate the eggs or vice versa, depending upon where we are in the kitchen. An informed observer would nevertheless view these behaviors as part of the overall ‘scramble eggs’ goal. An overly rigid prior specification of task steps could, in many instances, be quite counter-productive (e.g., the goal of ‘making a note of something’ should not fail because a particular pen and notepad are unavailable, when a pencil and paper serviette are to hand). Similar component steps can have quite different functions depending on the goal (e.g. picking up a saucepan to make scrambled eggs vs. using it as a paper weight).

Hence many have argued that goals are not simply a linguistic summary of the purpose of a series of actions, but reflect a unitary entity, a program, that coordinates relevant aspects of attention, cognitive control, mental representations (e.g. relevant memories) and actions towards the goal’s achievement (Anderson, 2014; Gollwitzer & Bargh, 2005; Greenwald, 1972; James, 1890; Jeannerod, 1988; Lewin, 1926; Meyer & Kieras, 1997; Prinz, 1987; Kruglanski & Kopetz, 2009). To take an example of a visual search for targets over a 20 s period. It certainly feels unlikely that this is instantiated by multiple second-by-second decisions to look for the target. Rather, there is a common program that establishes and maintains the relevant visual selectivity and response selection over the 20 s period. Were the task to be interrupted by a phone call, we would imagine a further planning step to re-establish the relevant program. Should the task change to responding to frequent ‘go’ targets whist withholding responses to rare ‘no-go’ stimuli, a new goal program with an additional inhibitory component would be established.

More generally, the program subsuming cognition during extended tasks may be about instantiating any goal-directed control that keeps cognition in the most optimal state achievable for the demands expected across the extended task. In more predictable tasks (e.g. during memorized task item executions, Schneider & Logan, 2006), the program may instantiate more detailed rule-related set changes in cognition across time (see also Logan & Gordon, 2001). In even more predictable tasks (e.g. memorized motor sequences) the program may even instantiate the component motor acts to be made across time and become the more famous motor programs (Keele, S. W., Cohen, A., & Ivry, 1990). Hierarchical and programmatic control of extended behavior may be a general principle of cognition.

This would predict that evidence of hierarchical control structures, previously seen during memorized task sequence execution, should be seen in all extended tasks, including those that are unpredictable and are not linked to any memorized sequence representation. To test this, Farooqui and Manly (2019) asked participants to execute rule-switch trials on which the relevant rule was signaled by the stimulus margin color that appeared with the stimulus onset (and hence could not be anticipated on any given trial). Participants saw two numbers displayed side-by-side at the center of the monitor and were asked to choose the smaller value when the outer margin color was blue and choose the smaller font when the margin color was green. Across consecutive trials, rules either stayed the same, or changed with equal probability. Crucially, through various means, participants were biased into construing 3 to 5 consecutive trials as one task-unit. For example, a number, irrelevant to the switch-task, counted-down from 3 to 1 in the background, or a slightly extended inter-trial interval was used to indicate the task-unit boundaries.

Even though the trials were standalone and unpredictable, and the e.g., 3-trial unit was merely a construed one, their execution showed the same 3 signs of hierarchical control structures that were previously seen during memorized task sequence executions. (1) RT on trial that began a task unit, i.e., trial 1 (or the 1^st^ step of the task unit), was the longest because a program had to be assembled at the beginning of the unit. (2) Since this program contained elements related to the entire task, and longer tasks require larger programs that take longer to assemble, trial 1 RT was longer before longer units. Further, such longer trial 1 RT was even seen before task units that were *expected* to have more, though uncertain, number of trials with unpredictable content (Experiment 5, Farooqui & Manly, 2019), as well as before task units that had the same number of trials but had a longer total duration (Experiment 6). Both suggesting that the program took longer to assemble when the ensuing task required longer or more complex attentional focus.

Switch cost, i.e., increased RT and error rates on switch compared to repeat trials, is related to a change in rule related sets or associations between the preceding and the current trials (Rogers & Monsell, 1995; Wylie & Allport, 2000). If programs were higher-level to individual trials, then configurations and associations related to individual trials will be nestled under them. Dismantling of these programs at task completion will also dismantle any underlying rule-related sets and associations leaving no disadvantage for switching rules. Hence, (3) rule-switch cost was absent on trial 1 (Farooqui and Manly, 2019). This account additionally predicts that trial-related demands that do not follow from an interaction between issues related to the preceding and the current trial, e.g., Stroop congruency effects, should not change at trial 1 of such task units. Unlike switch costs, the congruency effect in Stroop tasks (e.g., longer delays in identifying the font color of the word RED printed in black) follows largely from the incongruence between the different stimulus dimensions (i.e., word-meaning and font-color) of the *current* trial (MacLeod, 1991). Sequence effects on congruency (e.g., slower response on incongruent trials following congruent trials compared to incongruent trials following incongruent trials) are present but account for a very small fraction of congruency effects (Egner, 2007). Change in the overarching program should have no effect on congruency effects. This was indeed the case. Congruency effect or Stroop cost was unchanged at trial 1 (Farooqui & Manly, 2019, Experiment 4).

If programs are the means of executing a task unit, they would get assembled *prior* to trial 1 (or step 1) execution. Program instantiation therefore would not affect the actual demands of trial 1 execution. Trial 1 RT increases because it captures two sequential operations – instantiation of the program related to the larger task and actual trial 1 execution, and not because trial 1 execution became more demanding. This predicts that increased trial 1 RT should not be accompanied by increased error rates if there is no time pressure for its execution. This was indeed the case. RT on trial 1 was more than 150 ms longer than on subsequent trials but its error rates did not increase (Farooqui & Manly, 2019). In comparison, rule-switching was accompanied by significantly higher error rates even though it increased RT by only 50 – 100 ms.

### Programs and Cognitive Resources

When there are too many things to process or maintain, performance decreases, suggesting consumption of a limited ‘resource’ (Marois & Ivanoff, 2005; Norman & Bobrow, 1975) e.g., increasing the number of representations in WM above a certain point, decreases the fidelity of those representations (Ma et al., 2014). These limits are well known to constrain our capacity to process and maintain goal-directed operations and entities e.g., attention and WM items (Kahneman, 1973; Matthews, 2000; Wickens, 2008).

If programs are goal-directed entities that instantiate other control operations e.g., attention, and are maintained across extended tasks, they are likely to consume these resources and have a cognitive load, just like attention and WM. If programs embody executive commands for all control changes to be made across the task duration, then their cognitive load will be highest at the beginning of execution because programs, at this stage, have elements related to the entire duration of the ensuing task. As parts of the task get executed, elements related to the completed parts of the task would no longer be active, and the program load will decrease. Thus, the program load during a 40 s long attentional task will be highest at the beginning and will decrease as components of the task get executed. Further, the program load at the beginning of a 40 s long attentional task will be greater than at the beginning of 20 s long task.

In line with this, regions well-known to deactivate to cognitive load, e.g., the default mode regions (Raichle et al., 2001), show most intense deactivation at the beginning of execution when program load is likely to be the highest (Farooqui & Manly, 2018). This deactivation then reduces as parts of the task get executed and disappears by the last step. Furthermore, this deactivation is higher at the beginning of longer, compared to shorter, tasks. Likewise, pupil dilation, another measure of cognitive load, is larger at the beginning of longer versus shorter tasks (Unsworth & Robison, 2018).

### Our thesis

If larger programs have a higher load, then there will be limits on the size of program that can be instantiated/maintained. Programs related to very long tasks may not be maintainable and such tasks may not be executable as one unit, instead such tasks may have to be executed as a sequence of smaller units or chunks (or subtasks). A limit on the length of task executed as one unit has previously been characterized in situations where the task length was contingent on how much information could be kept in WM (see above, Logan, 2004; Bhandari & Duncan, 2014). If there are resource limits on program size, then such limits would be present even when the task is not limited by WM limits. When too long, such task would tend to get chunked into smaller units for their execution.

This thesis is also suggested by a famous observation about the nature of task labels that people employ to describe their behavior (Vallacher & Wegner, 1987). When executing easy and routine behaviors, individuals typically choose labels corresponding to the overarching sequence, for example, saying that they are ‘preparing breakfast’ rather than the subtask of ‘chopping fruit’. In contrast, when the same behavior in the same context becomes more challenging. e.g., when using a heavy and blunt knife, they are now more likely to use the ‘chopping fruit’ label. This suggests that the label reflects the length of behavior that gets executed as one task, and that this length is reduced during more challenging tasks, independent of WM limits. When chopping fruit with a very heavy knife, fewer resources are available to maintain programs and behavior gets executed as smaller task units through smaller programs. Likewise, as individual motor acts become easier and less demanding with practice, the size of the overarching program containing these motor components can increase (Ramkumar et al., 2016; Tosatto et al., 2022).

In all experiments here, we presented sequences made of eight or more demanding (e.g., rule-switch) trials and studied if these were executed through a single program or required a new program partway through the sequence. Our task was akin to Logan (2004), except that no memorized list was being executed across the trial sequence and the content of any trial could not be predicted. The item to be executed could only be known on that trial and would repeat or switch across consecutive trials with equal probability. The trial sequences were organized in the same way as in Farooqui and Manly (2019) – consecutive trials within a sequence had smaller inter-trial intervals (0.5 s), while those across sequences were separated by larger intervals (2 s; see methods for details). Since the trials were standalone and no information or representation linked trials of a sequence, even the idea that ten trials made a sequence was a construed one (see below for details). Only thing consistent across different iterations of these sequences was their consisting of a given number of trials. The actual content of what was executed on these trials would change unpredictably across consecutive sequences and were thus very variable both within and across participants. Such sequences of ~4 trials characteristically show the above-mentioned signs of program instantiation on trial 1 (Farooqui & Manly, 2019). We can expect the same here.

Our focus, however, was on trials 2 and beyond. If long trial-sequences could be executed by a single program, then the signs of program instantiation would be present only on trial 1 and not on subsequent trials. If the size of program was limited, and a single program for the entire sequence could not be maintained, then such long sequences will be chunked and executed through a series of smaller programs. The program instantiated at the beginning will only execute the first chunk and a new program will be needed for the second chunk and so on. In a 10-trial sequence (e.g.) the program instantiated at the beginning may only execute, say, trials 1 to 3, a second new program may execute trials 4 to 6, and the third one executes the rest. Program related signs, like RT peaks, will then be present not just on trial 1 but also on trials 4 and 7.

**Figure 1:**
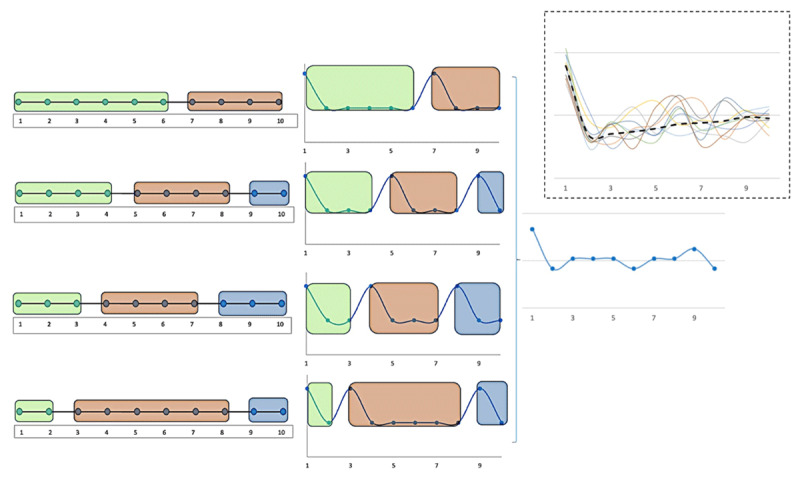
An illustration of a trial sequence execution that was chunked differently across four different iterations of execution. In the first iteration, trials 1 to 6 form chunk 1 and trials 7 to 10 form chunk 2. In the second iteration, the execution is parsed into 3 chunks (trials 1 to 4, trials 5 to 8 and trials 9 and 10). In the third iteration, trials 1 to 3, 4 to 7 and 8 to 10 form chunks 1 to 3. In the fourth iteration, trials 1 to 2, 3 to 8 and 9 to 10 form chunks 1 to 3. If RT peaks at the beginning of new chunks, then the RT pattern will vary across executions that are chunked differently, e.g., trial 7 will show a peak in iteration 1 but not in iteration 2 whereas trial 5 will show a peak in iteration 2 but not in iteration 1. Averaging such RT patterns will smudge the peaks. If the RT patterns are very variable, averaging will show a gradually increasing RT across trials 2 to 10 because the probability of having a peak increases with trial position.

Due to their inherent variability, RT patterns across trial-sequences can only be estimated after averaging performance over many iterations. The exact RT pattern seen when long trial-sequences are parsed and executed as a series of smaller chunks will depend on how the sequence was parsed. If, as in Logan (2004), a 10-trial sequence was parsed consistently into 3-trial long chunks, then program-related RT peaks will be present on trials 1, 4 and 7. But in Logan (2004), the same memorized sequence was iteratively executed and a discrete WM limit of 3–4 items ensured a constant chunk size (Cowan, 2001). But when trials of the sequence are demanding, and have variable and unpredictable task content, and are not constrained by a discrete WM limit, then parsing may vary across different iterations of execution. The pattern seen after averaging RTs from across several trial-sequence iterations will then depend on the range of possible chunk sizes and the probability of having them (Figure 1).

As a rule, if chunk sizes are small and variable, then RTs will show a general increase across the trial-sequence and will not show discrete peaks at specific positions (Figure 1 inset). This is because the probability of a trial beginning a new chunk and hence being the site of a new program instantiation will increase with its ordinal position. While trial 3 can only be a site for program instantiation when chunk 1 is two trials long, trial 8 can be a site in many more scenarios e.g., when chunk 1 is seven trials long, or when chunk 1 is two trials long and chunk 2 is five trials long, or when chunks 1 and 2 are three and four trials long etc. The number of possibilities that make later trials a site for a new chunk will always be higher than earlier trials. In case of variable sized chunking, averaging RTs over greater number of sequence executions will merge RT peaks such that the average pattern is one of gradual increase across the trial sequence (Figure 1 inset; See also Experiment 2 below).

This becomes apparent in a simulation. We generated expected RT patterns for 10-trial long sequences using data from Farooqui and Manly (Experiment 2; 2019). Programs in their study were instantiated on trial 1 and maintained across trials 2 to 5. We used the mean and standard deviation of their trial 1 RTs (1030 ± 46 ms) as the expected distribution of simulated trial 1 RTs as well as of trials 2 to 10 when these trials began a new chunk and hence were the sites of a new program instantiation. We used the means and standard deviations of trials 2 to 5 (850 ± 27 ms) from this study as the expected distribution of RTs on trials 2 to 10 when these were not sites of new program instantiation. We ran the simulation for 400 iterations across 16 hypothetical participants.

**Figure 2:**
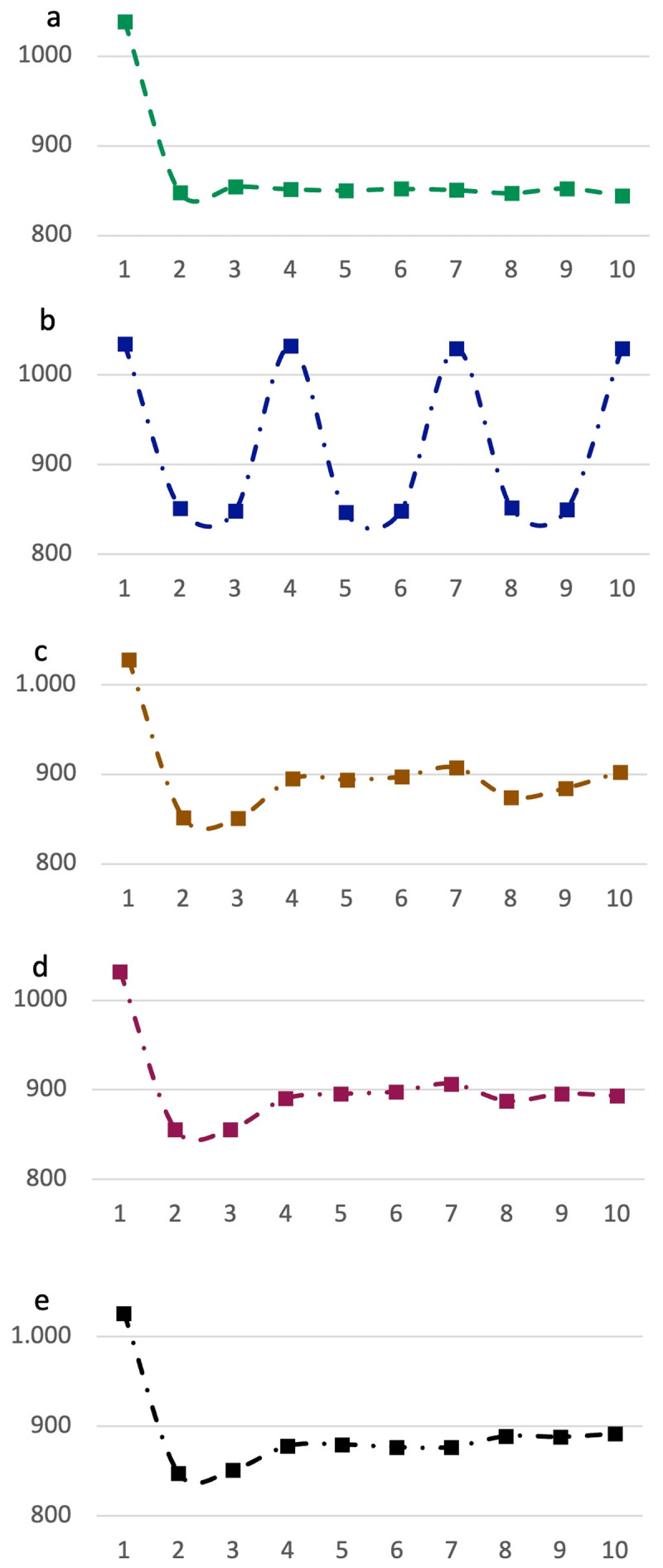
**(a)** If the entire sequence could be executed by the program assembled at trial 1, then trial 1 RT will be high but RTs across trials 2 to 10 would not differ. **(b)** If the sequence was always parsed into 3-trial chunks then RTs will additionally peak on trials 4, 7 and 10. But as chunk sizes become variable – 3 to 5 **(c)**, 3 to 6 **(d)** and 3 to 9 **(e)**, the pattern increasing becomes one of gradual increase across trials 2 to 10.

If resource limitations were not relevant and the program instantiated at trial 1 was sufficient for the entire sequence, then there would be no need for a re-instantiation on subsequent trials. The program related increased RT would only be seen on trial 1 (Figure 2a). RT on trials 2 to 10, in such a scenario, would not vary. Figure 2b shows the scenario when the sequence was chunked into constant 3-trial long units (c.f. Logan, 2004). RT peaks can be seen on trials 1, 4, 7 and 10. In the scenario where chunk sizes vary from 3 to 5 trials long with equal probability, then RTs show blunted peaks, with RTs increasing across all later trials (Figure 2c). If the chunk sizes varied from 2 to 6 (Figure 2d), or from 2 to 9 (Figure 2e), blunted peaks increasingly get replaced by a gradually increasing RT across trials 2 to 10.

Chunk sizes can of course vary in all kinds of ways not tested in these simulations both in their range and in their probabilities. Our aim in this study was primarily to see if execution of such long trial-sequences gets chunked. We predicted that if resource limitations limit the size of the program instantiated at trial 1, then (1) RTs across trials 2 to 10 would not be equal. They would either show a gradual increase or localized peaks or bumps or a combination (all of these would be consistent with our predictions). However, and crucially, if this increase in RT was caused by program instantiation, then trials with increased RT will also be accompanied by other program related signs (see above): (2) Error rates will not be increased on them; (3) rule-switch costs will be lower on them; and (4) Stroop incongruent cost will not be different on them.

## Experiment 1

### Methods

#### Participants and sample size calculation

Sixteen healthy participants (11 females; mean age 23 ± 3 years) were recruited. In this and all other experiments participants were recruited from the University of Cambridge MRC Cognition and Brain Sciences Unit healthy volunteer panel. This and all other experiments were approved by Cambridge Psychology Research Ethics Committee. In this and all other experiments, participants gave written, informed consent before the experiment, and were paid £8.50/session for their participation. All had normal or corrected to normal vision.

For calculating sample size, we did a pilot experiment that was identical to the rule-switching sessions of this experiment. It had 27 participants. We used its data to calculate sample size for detecting RT differences across trials 2–10 (see below) using GLIMMPSE software (https://glimmpse.samplesizeshop.org). This indicated that a sample size 16 participants would give power of 0.84 and type I error rate of 0.05.

#### Procedure

**Figure 3:**
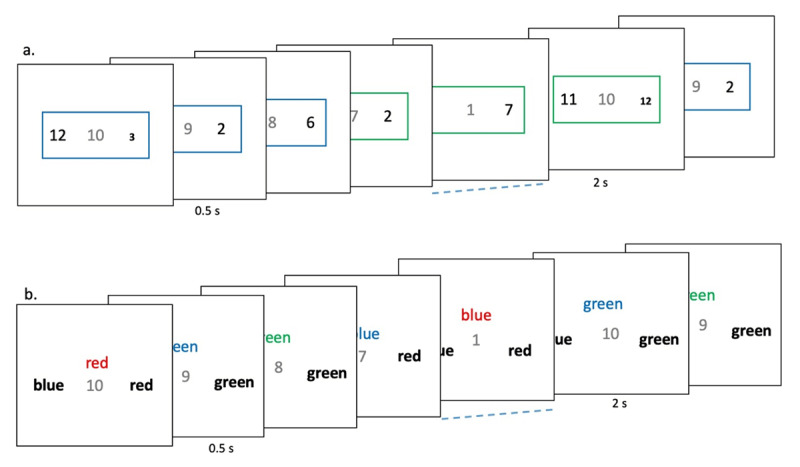
(Experiment 1) Participants executed 10 trial long sequences. Trials were organized into sequences by having low inter-trial intervals (0.5s) within a sequence and high inter-trial interval (2s) across sequence boundaries. This was further reinforced by a faded digit in the background that went from 10-9-8-…-1 across the 10 trials. **(a)** In Rule-Switch sessions, they executed sequences made of rule-switch trials. Trial rule was cued by the color of the outer margins: blue – choose the smaller value, green – choose the smaller font. **(b)** In Stroop sessions participants executed sequences made of Stroop trials where they chose the color of the print of the word from the two choices below.

Participants executed 10-trial long sequences. Trials within a sequence had smaller inter-trial intervals (0.5 s); the inter-sequence-interval was 2 s (Figure 3). An irrelevant number in the stimulus background counted down from 10 to 1 across the 10 trials of the sequence. The experiment consisted of two one-hour sessions done on different days.

One of the sessions, called the rule-switching session (Figure 3a), consisted of sequences made of rule-switch trials where the rule to be executed was conveyed by the color of the stimulus margins: blue – choose the smaller value of the two numbers, green – choose the smaller font. This colored margin came on at the same time as the stimulus. Across consecutive trials the probability of the rule repeating or changing was 0.5. These ensured that the rule to be executed on any trial could not be predicted beforehand.

On each trial of this rule-switching session, two numbers (between 0 to 99) differing in value and font size (Arial font 60 or 20) were displayed on each side of the center (Figure 3a). A faded digit (70% transparent) appeared at the center that conveyed the number of trials left in the current sequence. Trial 1 had the digit ‘10’ in the background, while trial 2 had ‘9’ and trial 3 had ‘8’, and so on. A blue or green square margin appeared around, and at the same time as, the number display, and conveyed the rule to be executed on that trial – blue: choose the smaller value, green: choose the smaller font.

On the other session, the Stroop session (Figure 3b), the same participants executed sequences made of 10 Stroop trials. Each trial had a color name printed in the same (70% trials) or different colored fonts (30% trials). Participants chose the color of the fonts from the two options below by making a button press. On each trial, participants saw a centrally presented color name (‘Red’, ‘Blue’, ‘Green’, and ‘White’; in Arial font size 40). It appeared in congruent font color on 70% of trials and in an incongruent color on the remainder (Figure 3b). On incongruent trials one of the choices always repeated the stimulus word. On congruent trials the incorrect word was selected randomly from the remaining colors. The actual order of these sessions was counterbalanced across participants.

In this and all subsequent experiments, participants’ decision was conveyed via button press that was spatially congruent with their choice (Numpad 1 for left and Numpad 2 for right on a standard QWERTY keyboard number pad), and the stimuli remained onscreen until a response was made. In all experiments, within a sequence, the next trial stimuli came 0.5 s after the response to the previous trial stimuli. After response to the 10th (i.e., the last) trial of a sequence, trial 1 stimuli of the new sequence came after 2 s. Erroneous responses in all experiments elicited a low-pitched feedback tone (70 ms in duration).

Before rule-switching sessions participants were given 7 practice trials on each of the two rules (value and font judgments). They were then told that the relevant rule will now randomly change and started session 1. Before Stroop sessions, the participants were told to chose from the two options presented below the color word, the one that corresponded to the color in which the word was written rather than the word’s meaning. In all experiments the participants were told that the number in the center that changed from 10 to 1 was irrelevant. Participants executed 200–300 sequences on each of the two sessions.

Experiments were created in Visual Basic.net and run on a Dell computer with an 85 Hz refresh rate monitor situated at a comfortable distance from the participant. The experiment was conducted on an individual basis in a testing room designed to minimize visual and noise distraction.

#### Analysis

For all experiments (unless otherwise specified) we did a repeated-measures ANOVA and a Bayesian ‘Repeated measures ANOVA’, both using JASP (2022; Version 0.16.3). For contrasts we used the polynomial contrast option in JASP and looked at linear contrast values. In all Bayesian analyses we used priors that were default to JASP which distributed priors uniformly across models. We use BF_10_ > 3 as evidence for the alternative hypothesis and BF_01_ > 3 as evidence for the null hypothesis.

In all our experiments, the initial 50 trials (or 5 sequences) were removed prior to analysis to minimize the influence of early practice effects, gaining familiarity with the responses etc. Error trials and trials with RTs outside 5 standard deviations (calculated individually for each participant) were also excluded from RT calculations.

### Results and Discussions

#### Rule-Switch Session

**Figure 4:**
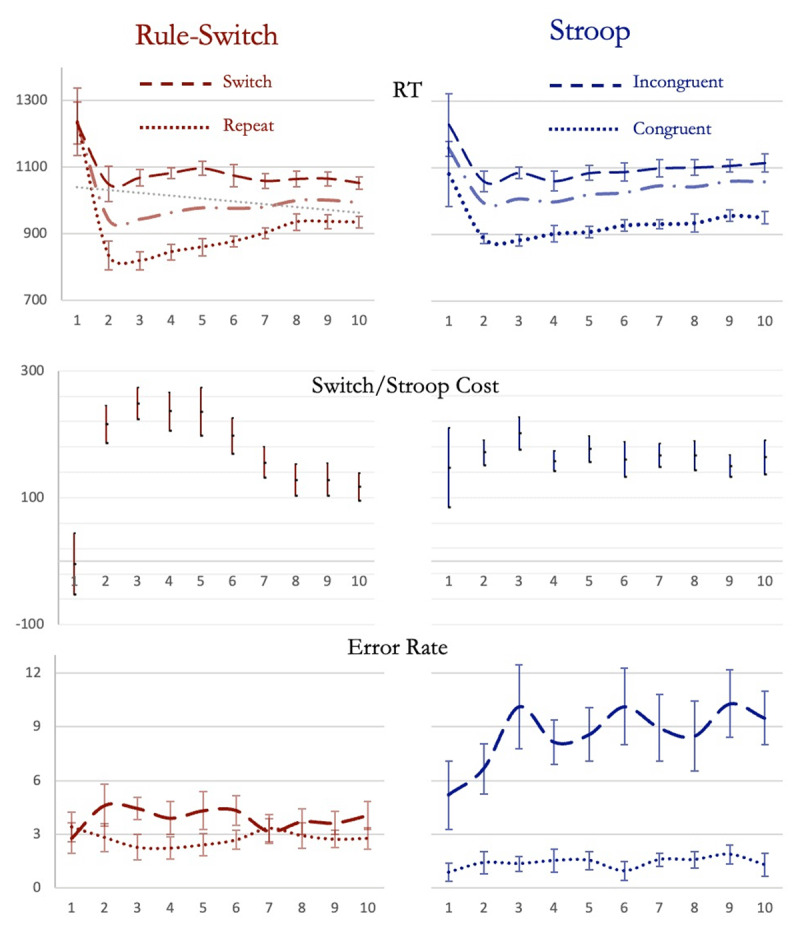
(Experiment 1) Reaction times, Costs (rule-switching and Stroop) and Error rates from rule-switching (left column) and Stroop (right column) sessions. Dashed lines represent RTs and error rates on rule-switch or Incongruent Stroop-trials. Dotted lines represent these during rule-repeat and Congruent Stroop-trials. The gray dashed and dotted line in top graphs represent averaged reaction times. Error bars in this and all subsequent graphs depict 95% confidence intervals.

We analyzed the data using both frequentist as well as Bayesian approaches. Trial 1, 2 …10 refers to the averaged correct RT for responses in those positions within the 10-item sequences (Figure 4, Left Column). We did a repeated-measures ANOVA on RTs with rule-switch and trial positions (2 to 10) as factors. The effect of trial position was significant (*F*_8,120_ = 3.7, *p* < 0.001, η^2^_p_ = 0.2) with a significant linear contrast across them (*t*_120_ = 5.1, *p* < 0.001), showing that RTs increased linearly across trials 2 to 10. The change in RT across trials 2 to 10 was different for switch and repeat trials (*F_8,120_* = 11.9, *p* < 0.001, η^2^_p_ = 0.4).

The same was affirmed in Bayesian analyses. The model that included switch-status (i.e., rule switch or repeat), trial position (2 to 10) and an interaction between them was significantly better in explaining the data compared to the null-model (BF_10_ = 4 × 10^14^). The Bayes Factor for including trial position effects in the model was 7.2 × 10^10^. In fact, even a model that only considered trial-position was better than null-model that included switch effects (BF_10_ = 22).

Crucially, unlike RTs, switch-cost decreased across trials 2 to 10. The effect of trial position on switch cost was significant (BF_10_ = 3 × 10^9^; repeated measures anova *F*_8,120_ = 12, *p* < 0.001, η^2^_p_ = 0.44; linear contrast: *t*_120_ = –9, *p* < 0.001). Furthermore, unlike reaction times, error rates were the same across trials 2 to 10 and did not change (BF_01_ = 105).

Others findings were expected from past studies. Trial 1 RT was higher than all other trials (*t* > 9, Holm-corrected *p* < 0.001, BF_10_ > 17650), switch trials had a higher RT than repeat trials (*F*_1,15_ = 47, *p* < 0.001, η^2^_p_ = 0.76, BF_10_ = 3839) and error rates (*F*_1,15_ = 21, *p* < 0.001, η^2^_p_ = 0.58; BF_10_ = 41). RT switch cost was absent at trial 1 (95% confidence interval: [–51 44]).

#### Stroop Session

As predicted, here too RT increased significantly across trials 2 to 10 (repeated measures anova *F*_8,120_ = 5.9, *p* < 0.001, η^2^_p_ = 0.28, linear contrast: *t*_120_ = 6.6, *p* < 0.001; Figure 4, right column). In Bayesian analysis, the model that considered effects of congruency and trial position (2 to 10) best accounted for the data (BF_10_ = 5.5 × 10^8^). Bayes Factor for including trial position effects in the model was 5248. Even a model that took only trial position (2 to 10) into account was markedly better than null model that included effects of congruence (BF_10_ = 5106).

Crucially, Stroop cost did not change across trials 2 to 10 (BF_01_ = 5.7). Switch and Stroop costs across trials 2 to 10 thus showed significantly different trends (BF_10_ = 4.3 × 10^9^). Unlike RTs, error rates were the same across trials 2 to 10 and did not change (BF_01_ = 15).

Again, trial 1 RT was expectedly the highest (*t*_15_ > 6, Holm-corrected *p* < 0.001, BF_10_ > 54). Stroop cost was not absent at trial 1 (*t*_15_ = 3.2, *p* = 0.006, 95% *CI*: [85 210], BF_10_ = 8.5). This was unlike the case with switch-cost (*t*_15_ = 3.2, *p* = 0.006, Cohen’s *d* = 0.8; BF_10_ = 8.6).

#### Summary

We thus found signs that long trial sequence was parsed and executed as small units. RTs on trials 2 to 10 were not equal but increased gradually towards trial 10. That this increase occurred because later trials were more frequent sites of program instantiation was suggested by an accompanying decrease in switch cost but with no change in Stroop cost or general error rates. If this increased RT was caused by continuous performance related issues like fatigue or loss of alertness, then it should have been accompanied by an increase in error rate, Stroop, and switch costs, reflecting a general depletion of resources and decrease in control abilities (Benoit et al., 2018; Kato et al., 2009).

Note that, unlike rule-repeat RTs, switch trial RTs did not increase across positions 2 to 10 (Figure 4). Two possible accounts are as follows. Potentially, in long trial-sequences that have a mixture of repeat and switch trials, *all* switch trials, and not just the later switch trials, start to serve as junctures for carving up the sequence into smaller chunks, and hence new programs were instantiated on all switch trials equally frequently. A second possibility is suggested by Altmann and Gray, 2002. Here, participants executed runs of 10–20 trials. The rule executed on any run was always constant, though it could change across different runs. Like us, they found that the RT increased across trials 2 onwards. They attributed this increase to an *adaptive* decay in the strength of rule representations across the length of the run to minimize interference with the new rule at the beginning of the next run. As per their account, in Experiment 1 here, the increase in rule-repeat RTs potentially occurred due to this kind of adaptive decay. By this argument, such an increase did not occur across rule-switch RTs because these trials, by definition, involve an updating of rule representations.

Experiment 2 arbitrates between these two accounts. Trial sequences here consisted only of rule-switch trials i.e., rules changed on every trial of the sequence. If the increase in rule-repeat RTs across positions 2 to 10 in experiment 1 was due to a decay in rule-representations (as suggested by Altmann & Gray, 2002) then such an increase in RT will not occur across trial sequences that consist only of rule-switch trials because rule-representations updated on every trial. In contrast, if this increase was due to later trials becoming more frequent sites of program instantiation, then later trials should show increased RTs because, since all trials are switch trials, they no longer are special junctures for carving up the long sequence. Now, the later trials will again be more frequent sites of new program instantiation. We also used Experiment 2 as an opportunity to look in detail at individual participant’s RT patterns. To power this analysis, rather than testing a large group of participants, a relatively small group completed many iterations of the task.

## Experiment 2

### Methods

Seven participants (4 females, mean age 22 years) executed 15-trial long sequences. In each sequence, the relevant rule (choosing the smaller value or the smaller font) alternated on every trial. The rule applying on trial 1 of each sequence also alternated between sequences, such that the value rule would appear on trial 1, 3, 5… in one sequence and trial 2, 4, 6 … of the next. The frequency of the two rules being executed on any trial position was thus identical. Participants did two sessions on different days, each lasting around 1.5 hours, and executed a total of 240–350 sequences. Other aspects of this experiment were identical to the Rule-Switch Session of Experiment 1. With this design, we hoped to get reliable estimates of RT pattern in every single participant. We therefore tested only seven participants since we expected significant results in every one of them.

### Results and Discussion

**Figure 5:**
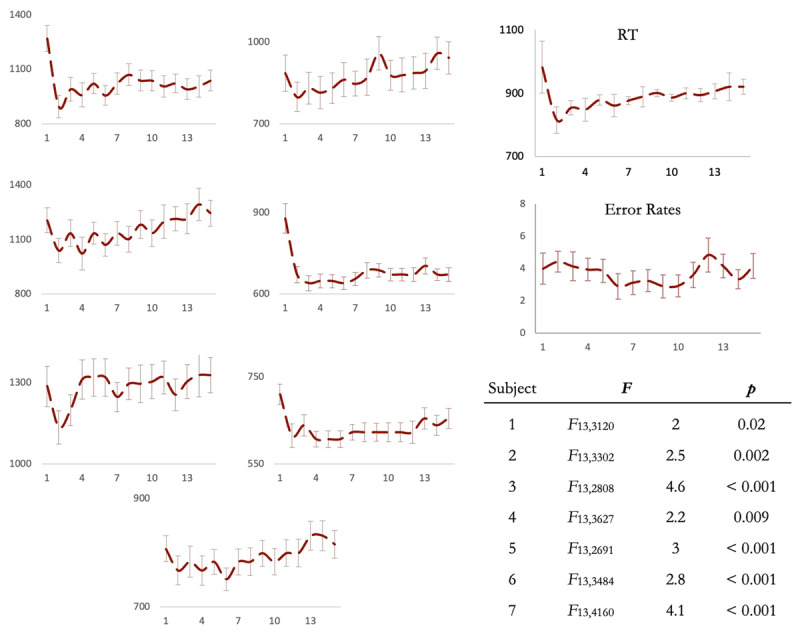
(Experiment 2) RT patterns across the seven participants. In each participant, RTs across trials 2 to 15 were significantly different (Table). The RT pattern after averaging across all participants (inset) was identical to that in Experiment 1. Error bars represent 95% confidence intervals.

RTs across trials 2 to 15 were significantly different in every single participant (Figure 5, table). While the RT pattern differed across the seven participants, the averaged RT pattern was identical to that seen in experiment 1 (Figure 4 inset; *F*_13,78_ = 4.4, *p* < 0.001, η^2^_p_ = 0.42, linear contrast: *t*_78_ = 7, *p* < 0.001; BF_10_ = 1080). Thus, the increase in RT on later positions of trial-sequences was present even when the rule representations were updated on every trial. This suggests that decay of rule-representations across time cannot be an explanation. Averaged error rates were not constant across trials 2–15 (*F*_13, 78_ = 2.4, *p* = 0.01, η^2^_p_ = 0.29; BF_10_ = 5.8) but, unlike RTs, however, did not show a systematic increase from trial 2 to trial 15 (*t*_78_ = –0.07, *p* > 0.9); trials with the longest RTs were not those with the highest errors.

Note that these results also illustrate the point that we made earlier. As apparent in Figure 5, the actual RT pattern was different across different participants. However, averaging them created a pattern of gradually increasing RT across the trial sequence.

## Experiment 3

We have argued that the gradual increase in RT across the length of the trial-sequence in Experiments 1 and 2 was caused by its variable-sized chunking that made later trials more frequent instances of new program instantiation. This predicts that, if instead of leaving it to spontaneous parsing we are able to bias participants into parsing a long sequence into *specific*-sized chunks (e.g. 3-trial chunks), the program onset related RT peaks will occur on specific trial positions (1, 4, 7, 10 and so on) and the gradual systematic increase in RT across *all* trials from trial 2 onwards will be abolished. The current experiment tested this thesis. We biased participants into chunking a long trial sequence consistently into either 3-trial long chunks or 5-trial long chunks.

### Methods

**Figure 6:**
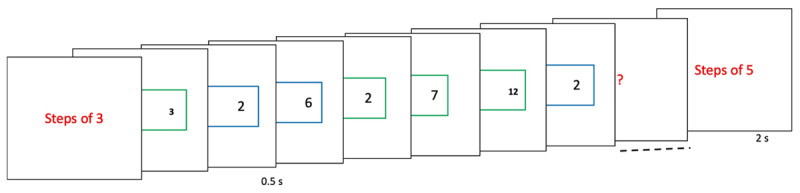
(Experiment 3) Participants were biased to chunk the larger task episode, consisting of up to 20 trials, into specific sized sub-tasks by making them keep a count of the trials in 3s or 5s (informed by the cue screen at the beginning).

The experiment design was identical to Experiment 1 of Farooqui and Manly (2019). But instead of analyzing RTs according trial positions within a chunk as they had done, we averaged RTs based on trial positions in the larger sequence. This would mean we would get fewer repetitions of events of interest per participant. We therefore took double the number of participants as the original authors. Thirty-one participants (18 females; mean age 21.6 ± 2.3 years) executed switch trials identical to that in experiment 1 (Figure 6). These were organized into 10 to 20 trials long sequences, the actual length being unpredictable. Crucially, we attempted to bias participants into parsing these long sequences into specific sized chunks by making them keep a covert count of these trials in threes (123123…) or fives (1234512345…). The sequence began with an instruction screen informing whether the count was to be in threes or fives. The sequence would terminate with a probe asking participants to key in the count of the trial they had just executed. For example, if a sequence being parsed into 5 item chunks was stopped after 18 trials, the correct answer to the probe should be 3 (12345 12345 12345 123). Participants executed 80 sequences in a session. Other aspects of this experiment were identical to Experiment 1.

If the gradual increase in RTs seen in experiments 1 and 2 was related to fatigue, waning attention etc. then RTs in the current experiment should similarly systematically increase across the long trial sequences. In contrast, if that increase had resulted from the averaging of program related RT peaks that occurred with greater frequency on later trials, as we have suggested, then biasing participants to parse the long sequence into specific-sized chunks will create program related peaks at those positions (e.g., every 4th trial when counting in 3s) and will abolish the gradual increase in RT across the sequence.

### Results and Discussion

**Figure 7:**
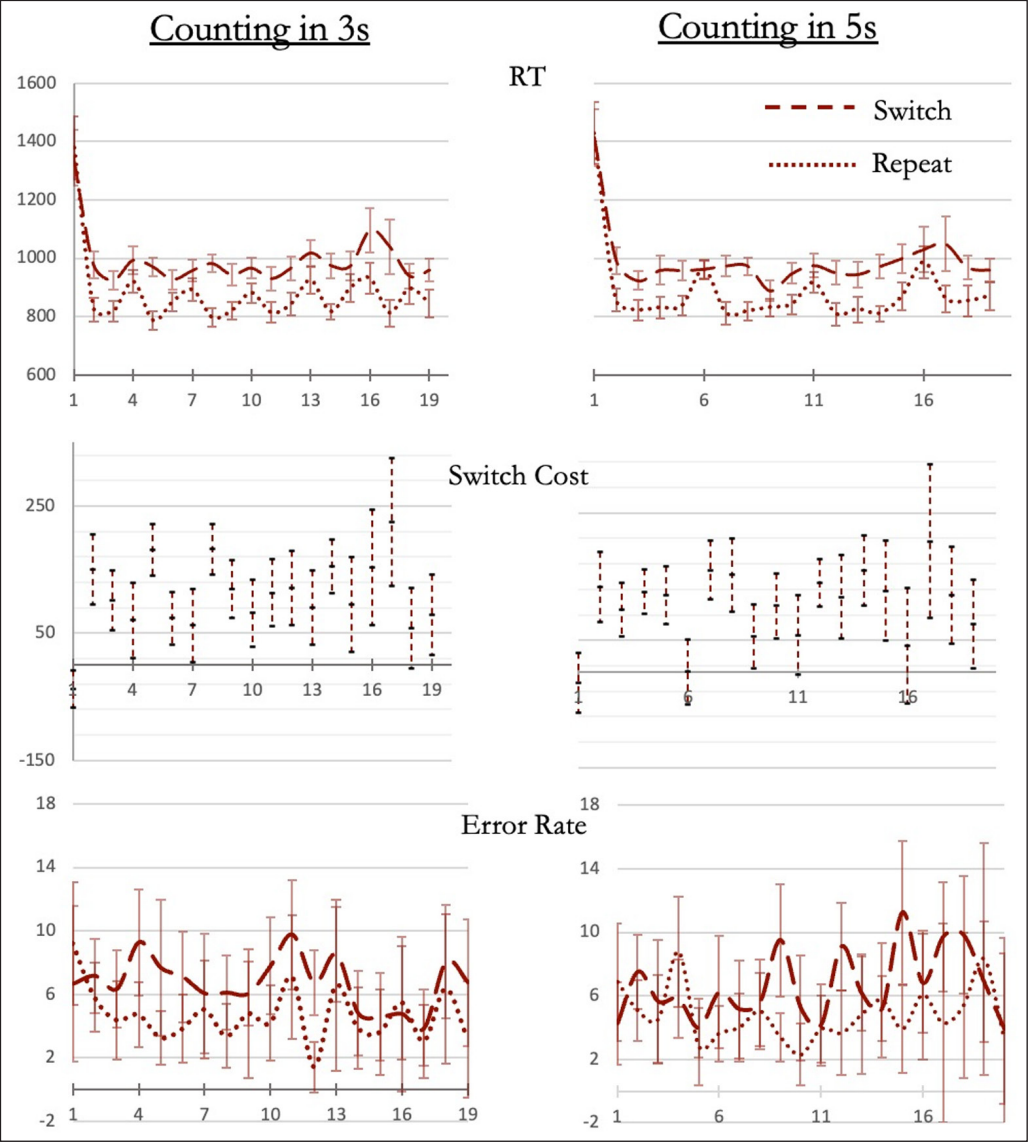
(Experiment 3) RT, switch cost and error rates across the trials of the sequence (rule-switch trials: dashed lines; rule-repeat trials: dotted lines). Error bars: 95% confidence intervals.

We limited our analysis to trials 2 to 15 because sequences longer than 15 trials were relatively rare, so the variability of RTs on trials beyond trial 15 was much higher. Our model looked at the effects of trial position and rule-switching on RTs. Across trials 2 to 15 of both sequence types, RTs varied significantly (Figure 7; 3-trial sequences: *F*_13, 377_ = 5, *p* < 0.001, η^2^_p_ =0.15, BF_10_ = 25667, BF for including trial-position = 10^6^; 5-trial sequences: *F*_13, 390_ = 4.7, *p* < 0.001, η^2^_p_ =0.14, BF_10_ = 1762; BF for including trial-position = 57829). But, crucially, unlike experiments 1 and 2, the linear contrast across trials 2 to 15 was not significant (3-trial sequences: *t*_29_ = 1.6, *p* = 0.2; 5-trial sequences: *t*_30_ = 0.002, *p* = 0.9) showing that RTs did not show a general increase.

Further, if the effect of trial position in the above analysis was driven by RT peaks on trial positions starting a new chunk (i.e., trials 4, 7, 10 and 13 in 3-trial sequences; trials 6 and 11 in 5-trial sequences) then removing these trials from the above analysis will remove the effect of trial position on RTs. This was indeed the case. With these trials removed, trial position had no effect on RTs and the null model became the more probable model (3-trial sequences BF_01_ = 9.4; BF for excluding trial position from the model = 9.9; 5-trial sequences: BF_01_ = 5.5; BF for excluding trial position from the model = 7.9). Furthermore, for trials between positions 2 and 15, RTs were higher on those that corresponded to a chunk position (e.g., trials 4, 7 and 10 for 3-trial sequences) than those that did not (e.g., trials 2, 3, 5, 6, 8 and 9 for 3-trial sequences; *t*_30_=5.6, *p* < 0.001, Cohen’s *d* = 1; BF_10_ = 4419). Again, unlike RTs, error rates did not vary and were the same across these trials (3-trial sequences: BF_01_ = 42; 5-trial sequences: BF_01_ = 1339).

Thus, biasing participants into chunking the long sequence into specific-sized units caused RT peaks to occur at specific junctures and removed the gradual increase in RT across the length of the sequence. Gradual increase in RT across a long trial sequence seen in experiments 1 and 2 therefore cannot be related to the effects of continuous performance and was likely due to variable sized chunking of the long sequence.

## Experiment 4

Cognitive resource limits on the size of the subsuming programs lead to a counterintuitive prediction. Preparation effects, whereby long and/or complex tasks have longer step 1 RT, are well known (e.g., Farooqui & Manly, 2019; Schneider & Logan, 2006). Hence, tasks consisting of 5 trials have higher trial 1 RT than those with 3 trials (Farooqui & Manly, 2019), and memorized sequences with more task item switches have higher trial 1 RT than those with fewer switches (Schneider & Logan, 2006). These show that longer/complex sequences require larger/complex programs that take longer to assemble, causing longer trial 1 RT. Such findings, however, come from sequences that were short and simple enough to be executed via a single program that, by inference, did not reach capacity limitations.

Preparation effects may be different when sequences to be executed are so long that program for the entire sequence cannot be instantiated/maintained at a time. The actual size and coverage of the program instantiated at trial 1 of such sequences will depend on the availability of cognitive resources. During sequences made of more demanding trials (e.g., those involving rule-switches) less resources will be available for the program because more resources will get used for executing individual component trials. In comparison, during sequences made of easier trials (e.g., those involving only rule-repeat trials) more resources will be available for the program because less will be needed for executing component trials. Consequently, larger programs may be maintainable during long and easy trial-sequences compared to during long and difficult ones. Hence, larger programs may be get assembled at the beginning of *long and easy* trial-sequences causing *larger* delays in trial 1 RT. In comparison, the program assembled at the beginning of *long difficult* trial-sequences may be of smaller size and cause *shorter* delays in trial 1 RT. In other words, it may paradoxically take longer to begin executing long sequences made of easy trials than long sequences made of difficult trials.

Relatedly and following the logic outlined in the previous experiments, if easy tasks can be executed via a *single* program from trial 1-n, we would not expect the cumulative increase in RT over those trials. In contrast, if difficult tasks require more programs, the cumulative increase in RT towards later trials of the sequences, seen in the previous experiments, will be observed.

### Methods

**Figure 8:**
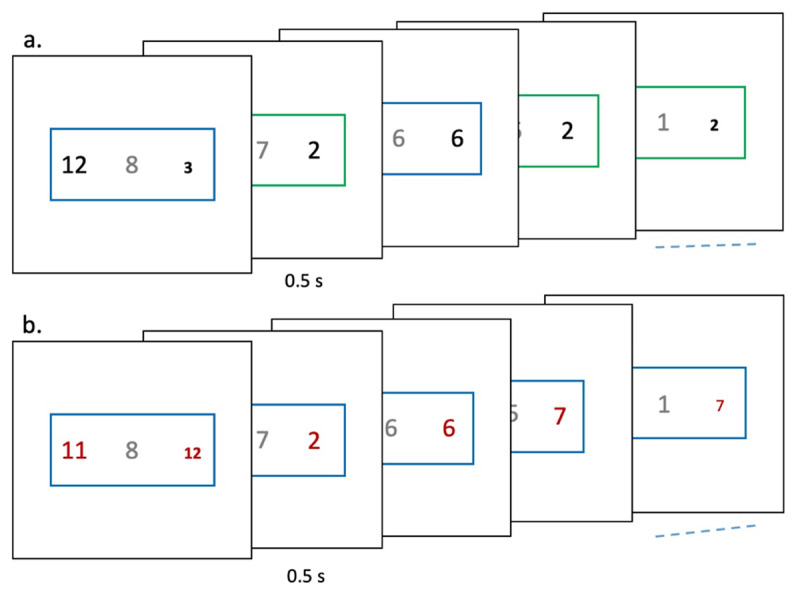
(Experiment 4) Two kinds of trial-sequences were executed. **(a)** Difficult sequences consisted of rule-switch trials whereby rules could change across trials. Number stimuli here were of black color. **(b)** Easy sequences consisted of only rule-repeat trials. Here the number stimuli were crimson in color.

The sample size was calculated using Farooqui and Manly’s (2019) data. We combined all their experiments that had long and short episodes. We assumed that the effect size on trial 1 RTs from instantiating large vs small programs would be similar. We used GLIMMPSE software. It indicated that twenty-one participants would give us a power of 0.9 and type 1 error rate of 0.05. We recruited twenty-three participants (8 females; mean age 23.3 ± 2.4 years) who executed two kinds of 8-trial long sequences (Figure 8). In the difficult task, the relevant rule (conveyed by the stimulus margin color) switched between choosing the smaller value of the two numbers and choosing the smaller font. The number stimuli during these sequences were presented in black fonts. In the easy task, the trial rule remained constant and hence, there was no switching. Here the number stimuli were presented in crimson fonts. The number font-color thus informed the participant (at least implicitly) whether the current sequence was difficult or easy. Individual trials were organized into 8-trial long sequences in the same way as in experiments 1. Stimuli and trial rules were identical to previous experiments. In fourteen participants, rules changed on each trial like experiment 2. In the rest of the participants, rules changed with a probability of 0.5 like experiment 1. The actual rule executed on trial 1 of the two sequences was precisely matched within each participant. The mapping between the font-color and sequence type was balanced across participants. The two kinds of sequences were randomly interleaved. Participants executed a total of 200–300 sequences.

### Results and Discussions

**Figure 9:**
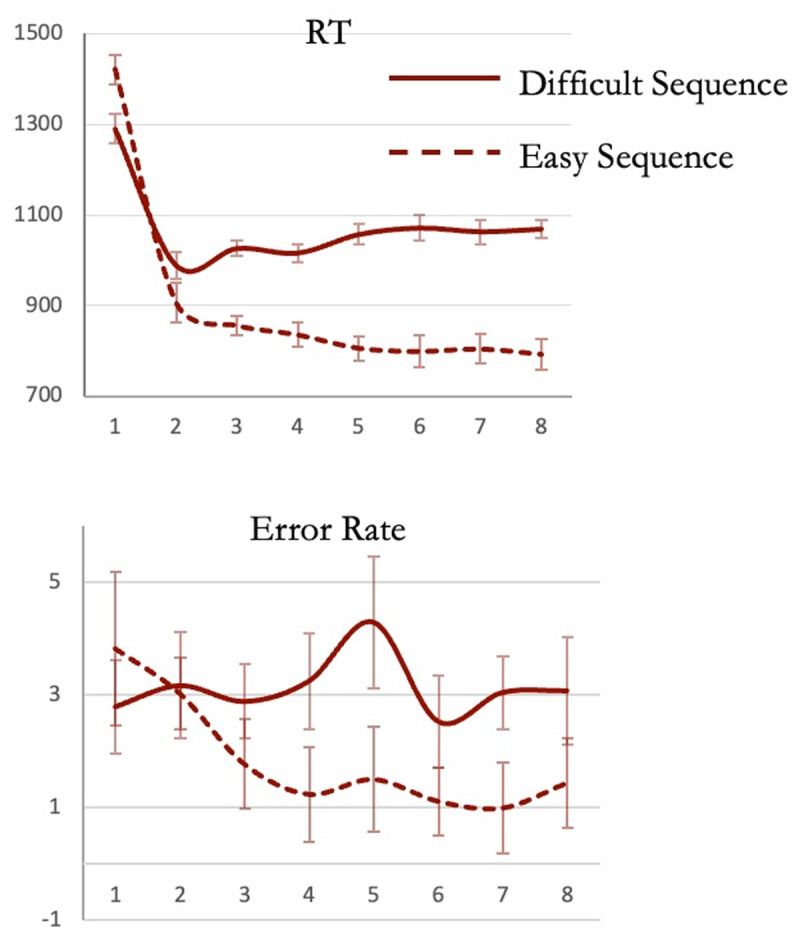
(Experiment 4) Continuous lines depict performance during difficult sequences made of rule-switch trials, dashed lines depict performance during easy sequences made exclusively of rule-repeat trials. As predicted, trial 1 RT was paradoxically higher for the easy sequences, while RTs on trials 2 to 8 were expectedly higher for difficult sequences.

Confirming our hypotheses, RT during trial 1 of easy sequences was indeed *slower* than during trial 1 of difficult sequences (Figure 9; paired *t*_22_ = 3.9, *p* < 0.001, *CI* of difference = [61 200], Cohen’s *d* = 0.81; BF_10_ = 43.4), suggesting that the program instantiated at the beginning of easy sequences was indeed larger than that instantiated at the beginning of difficult ones.

Expectedly, RTs on trials 2 to 8 of easy compared to difficult sequences were faster because the former were always rule-repeats while the latter involved rule-switches (*F*_1, 22_ = 46, *p* < 0.001, η^2^_p_ = 0.7, BF_10_ = 16225). RTs during difficult sequences increased across trials 2 to 8 (*F*_6,132_ = 6.71, *p* < 0.001, η^2^_p_ = 0.23; linear contrast: *F*_1,22_ = 18, *p* < 0.001; BF_10_ = 5170). In contrast and crucially, during the easy sequences, not only did RTs not increase across these trials, but they *decreased* (*F*_6,132_ = 9.4, *p* < 0.001, η^2^_p_ = 0.3; linear contrast: *F*_1,22_ = 16, *p* < 0.001; BF_10_ = 850468). The difference between the trends shown by trials 2 to 8 of difficult and easy sequences was thus significant (*F*_6,132_ = 18, *p* < 0.001, η^2^_p_ = 0.45). In Bayesian analysis comparing the two sequences, not only was the model that included effects of sequence type, trial position and an interaction between them the best (BF_10_ = 2.2 × 10^17^), the BF for including an interaction between them in the model was 5 × 10^13^.

Errors again did not change across these trials 2 to 8 of difficult sequences (BF_10_ = 1.3; linear contrast *t*_132_ = –0.37, *p* = 0.7), however, error rates *decreased* across these trials of easy sequences (BF_10_ = 24, linear contrast *t*_132_ = –3.5, *p* < 0.001). Both RT and error rates suggesting that the easy sequences were not chunked into smaller units and instead were executed as one whole through a single program.

This decrease in RT and error rates across easy trial sequences may have occurred because of the decrease in program load as parts of the task were executed. At trial 2 the program being maintained would have elements related to the remaining 7 trials, while at trial 5 it would have elements related to only 4 remaining trials. Consequently, as the sequences were executed, more and more cognitive resources became available for the execution of individual trials and hence the faster RT and decreased error rates.

## General Discussion

### Summary

It is well known that extended task execution begins with the instantiation of higher-level programs (e.g., Farooqui & Manly, 2019; Schneider & Logan, 2006). Here we presented evidence of such programs getting instantiated partway during the execution of long trial sequences, evidencing that the program instantiated at the beginning could not execute the entire sequence. We suggested that such trial sequences were chunked into smaller units and executed piecemeal. Note that the trial sequences were not merely cut but were chunked because each smaller *chunk* was covered by a subsuming program.

Chunking of a larger sequence into smaller units has previously been characterized in the context of memorized task sequences e.g., memorized long task-lists are recalled in WM as small chunks (Anderson et al., 1998; Logan, 2004). Trial sequences in the current study, however, did not involve recollection from memory, hence their chunking was not related to WM limits. Further, while the WM chunks of previous studies had a near-constant size and could hence be located by discrete RT peaks, chunks in the current study likely varied a lot in their sizes due to e.g. individual differences. This meant that later trials of the sequence were more frequent sites of program instantiation, and hence contained more RT peaks related to new program instantiation (Figures 1 and 2). Averaging RTs across many sequences created a pattern of gradual increase.

This was evidenced by the presence of other program related signs – decreased switch costs, unchanged error rates and Stroop costs and, critically, by the results of Experiment 3. When we biased participants into chunking long trial-sequences into specific-sized units (e.g., 3-trial long chunks or 5-trial long chunks), the gradually increasing RT pattern was replaced by RT peaks at specific positions at the beginning of the instructed chunks. This also ruled out any effect of fatigue, distraction etc., resulting from continuous performance as an explanation for increased RT on later trials of Experiments 1 and 2 because now participants executed very long trial sequences with no increase in RT on later trials.

### An Alternative Explanation

Perhaps maintaining the program over time gradually depleted cognitive resources and caused a gradual increase in RT. Resource depletion was primarily related to the duration for which the program had been maintained. This account has the advantage of explaining not just the increase in RTs in experiments 1 and 2 but also their absence in experiment 3. The program in experiment 3 was only maintained for 3 or 5 trials and participants got regular ‘rest-breaks’.

In essence, this explanation is in line with our thesis. It points to resource limits related to the maintenance of goal-directed program whereby maintaining them across time depletes cognitive resources. But this explanation cannot explain results related to error rates, switch, and Stroop costs. Depleting resources should increase both switch and Stroop costs, since difficult trials being more resource-dependent should slow down more and have more error rates later in the sequence. Furthermore, since this account focuses on resource depletions that come about from maintaining programs across time, it cannot explain experiment 4 results that suggested resource limitations affect the size of the program assembled at the beginning of trial-sequences.

### Programs

Hierarchical execution is a well-known principle in predictable motor and task sequences wherein the programs related to the ensuing tasks are instantiated at the beginning of execution. These then subsume the execution and are thought to specify the identity and sequence of component acts (e.g., Dezfouli et al., 2014). Current and related studies (Farooqui & Manly, 2018, 2019) suggest that this may be a more general principle of cognition and occurs during all kinds of tasks.

What would be the role of these programs? We suggested any task related control that is to be applied across task duration e.g., attention, may be instantiated via such programs. Additionally, this program may coordinate *relevant* aspects of attention, cognitive control, mental representations (e.g. relevant memories) and actions towards the goal’s achievement. In predictable tasks e.g., memorized task sequences (e.g., Schneider & Logan, 2006) these programs may *additionally* instantiate the relevant rule-related set related to each component step. In more predictable tasks, like practiced motor sequences (e.g., Rosenbaum et al., 1983), these programs may even specify the motor acts to be made across the task duration and become motor programs (Keele, S. W., Cohen, A., & Ivry, 1990).

The current study suggested a limit on the magnitude of control commands that can be embodied in a program. For long demanding tasks, a single program that will control the entire execution may not be instantiable or maintainable. Such tasks are instead executed as a series of smaller tasks (or subtasks). Note that this limit is not determinate in terms of number of trials or steps making up a task. In Experiment 4, long easy sequences could be executed in their entirety without any sign of program instantiation later in the sequence. How long a task can get executed via a single program may depend on its difficulty.

### Programs and Working Memory

Both working memory and programs are task-related and goal-directed but temporarily maintained entities. However, WM is typically used to refer to task-related declarative representations (Baddeley, 2012; Cowan, 2016). Programs in the experiments here and elsewhere (e.g., Farooqui & Manly, 2019) were not made of such declarative representations. While programs and working memory are clearly not synonymous, the two may be related. If programs are the means of instantiating all kinds of goal-directed processes during task execution, they may also be the means of maintaining task-related working memory representations across time. This is suggested by an observation in Schneider & Logan, 2006. Here, participants executed a memorized 4-item list iteratively. RT was highest for item 1. Since it was the same list being iteratively executed, this increased RT could not have been due to participants forgetting and then having to re-recall the list at the beginning of each iteration. It’s more likely that the increased RT resulted from the requirement to integrate the list representation present in memory into the new program being assembled at the beginning of each iteration. Perhaps this integration of mnemonic representations into the currently active program changes memory into *working* memory. Task relevant representations during execution may be maintained via their incorporation into the goal-directed programs. This would predict that program limitations described in this study may be related to the well-known working memory capacity limitations.

### Programs and Attention

Top-down attention creates a hierarchically organized cognition (Broadbent, 1977). The higher-level goal-related cognitive entities bring about attention-related changes in the lower-level cognitive processes and facilitate goal-relevant processes over those that are goal-irrelevant (Desimone & Duncan, 1995). During extended tasks such top-down attention must be instantiated and evolved across time such that, at every instance of the task, the most goal-relevant operation is favored. Compared to a short instance of attention that just requires the right cognitive focus, sustained attention across extended tasks additionally requires maintaining, and perhaps evolving, the attentional focus across time such that at each instance cognition is in the most optimal state achievable. The executive commands that bring about such attentional changes across time are unlikely to be launched as independent acts at every successive instance. It is more likely that during extended tasks, attentional changes across time are instantiated through a common program assembled at the beginning, much like how a single motor program brings about the various component motor acts across time.

The current study suggests that limits in sustaining attention across time may, at least partially, be related to the limits on how long a period of cognition is to be organized as a single task unit. Sustained attention during long tasks may be poorer for many reasons: (1) The load of the program subsuming a long task may be high and deplete the resources available for executing component acts. (2) Later steps of a long task may be more frequent sites of new program instantiation. This will not only increase the RT on them but make also make them error-prone if (unlike the current experiments) the time available to execute component steps is limited. A part of this limited time will then get used in instantiating the new program, leaving less time for executing the component step. Lastly, (3) it is also possible that maintaining program across time is effortful, and so becomes more and more difficult with time.

### Chunking of Control

Chunking is a well-known principle of cognition and can be seen in perception (Zacks & Tversky, 2001), memory representations (Bousfield & Bousfield, 1966; Miller, 1956) and actions (Diedrichsen & Kornysheva, 2015; Henry & Rogers, 1960). Even sets of processes during early perceptual processing (Cavanagh et al., 2001; Ullman, 1984), as well as those that occur later during the formation off a goal relevant conclusion about perception (Logan, 1999), may get instantiated as one chunk. Constructs about task related knowledge through which behavior is executed and controlled, e.g., schema, plans, scripts, productions, also invoke the idea of chunks (Cooper & Shallice, 2000; Miller et al., 1960; Schank & Abelson, 1977). Chunks across these diverse situations consist of a specific and concrete sequential structure. Instantiating such chunks instantiates both the identity and the sequence of its components. Such chunks, therefore, are seen to develop with practice and expertise in routinized situations (Gobet & Simon, 1998).

In comparison, chunks in the current experiments cannot be characterized as a set of linked representations having a determinate sequential structure. They corresponded to a set of unpredictable and unknown trials that were controlled and executed as one unit. Previously, even tasks consisting of an uncertain number of trials and framed as one entity, were executed, and controlled as one unit (Experiment 4, Farooqui & Manly, 2019). Current study suggests that chunks primarily are the episodes of cognition controlled as one unit. Past chunk characterizations are special cases of this general phenomenon. Mnemonic chunks are memories that can be recalled as one unit, motor chunks are acts that can be instantiated as one unit.

### An Unrecognized Aspect of Cognitive Resource Limits

Resource limitation is a key feature of cognition. When we have to deal with too many things — attend to many events, perform many tasks, keep lots of items in working memory — our performance declines, suggesting that some limited cognitive resource has become too distributed, leading to inefficient functioning. These resources are abstract and domain-general and can be allocated in parallel to a wide range of cognitive functions and processes. Past work has characterized resource limitations across many situations (Marois & Ivanoff, 2005; Norman & Bobrow, 1975). Working memory limits are seen when people simultaneously keep a lot of representations (like words or visual objects) in mind while also executing other tasks (Cowan, 2016). Attentional limits are seen when more than one object or space in the environment has to be simultaneously attended to (Franconeri, 2013). Similar limits also constrain the capacity to simultaneously proceed with more than one task (Pashler, 1994). In all such scenarios resource limitations limit the ability to do things simultaneously. In contrast, we evidence a new aspect of resource limitation that limited the length of behavior that can be executed sequentially as one unit. This limitation may be one of the reasons why we frequently chunk long tasks into subtasks (Bandura & Schunk, 1981). Recognition of this limitation will enhance our understanding of diverse aspects of cognition including sustained attention, working memory and chunking.

## Data Accessibility Statement

Data can be accessed from this link: https://osf.io/89ky2/?view_only=33145dac3b7641d8819c172893338770.
